# Endoscopic Treatment of Biliary Stenosis in Patients with Alveolar Echinococcosis – Report of 7 Consecutive Patients with Serial ERC Approach

**DOI:** 10.1371/journal.pntd.0004278

**Published:** 2016-02-24

**Authors:** Marija Stojkovic, Thomas Junghanss, Mira Veeser, Tim F. Weber, Peter Sauer

**Affiliations:** 1 Section Clinical Tropical Medicine, University Hospital Heidelberg, INF 324, Heidelberg, Germany; 2 Interdisciplinary Endoscopy Unit, University Hospital Heidelberg, Department of Internal Medicine, INF 410, Heidelberg, Germany; 3 Department of Diagnostic and Interventional Radiology, University Hospital Heidelberg, INF 410, Heidelberg, Germany; Universidad Peruana Cayetano Heredia, PERU

## Abstract

**Background and Aims:**

Biliary vessel pathology due to alveolar echicococcosis (AE) results in variable combinations of stenosis, necrosis and inflammation. Modern management strategies for patients with cholestasis are desperately needed. The aim is proof of principle of serial ERC (endoscopic retrograde cholangiography) balloon dilation for AE biliary pathology.

**Methods:**

Retrospective case series of seven consecutive patients with AE-associated biliary pathology and ERC treatment in an interdisciplinary endoscopy unit at a University Hospital which hosts a national echinococcosis treatment center. The AE patient cohort consists of 106 patients with AE of the liver of which 13 presented with cholestasis. 6/13 received bilio-digestive anastomosis and 7/13 patients were treated by ERC and are reported here. Biliary stricture balloon dilation was performed with 18-Fr balloons at the initial and with 24-Fr balloons at subsequent interventions. If indicated 10 Fr plastic stents were placed.

**Results:**

Six patients were treated by repeated balloon dilation and stenting, one by stenting only. After an acute phase of 6 months with repeated balloon dilation, three patients showed “sustained clinical success” and four patients “assisted therapeutic success,” of which one has not yet reached the six month endpoint. In one patient, sustained success could not be achieved despite repeated insertion of plastic stents and balloon dilation, but with temporary insertion of a fully covered self-expanding metal stent (FCSEMS). There was no loss to follow up. No major complications were observed.

**Conclusions:**

Serial endoscopic dilation is a standard tool in the treatment of benign biliary strictures. Serial endoscopic intervention with balloon dilation combined with benzimidazole treatment can re-establish and maintain biliary duct patency in AE associated pathology and probably contributes to avoid or postpone bilio-digestive anastomosis. This approach is in accordance with current ERC guidelines and is minimally disruptive for patients.

## Author Summary

Alveolar echinococcosis (AE) is a zoonosis causing infiltrative liver lesions. A subgroup of patients presents with central liver lesions and biliary obstruction. At present there is no clear concept for the treatment of biliary obstruction in AE of the liver, and data from high quality trials to base treatment decisions on evidence are missing. In rare neglected infectious diseases with very low prevalence, clinical data can mainly be generated from case series. In our study we aim at the proof of principle of serial ERC balloon dilation for biliary pathology associated to alveolar echinococcosis. This approach is in accordance with current ERC guidelines and is minimally disruptive for patients.

## Introduction

Alveolar echinococcosis (AE) is a parasitic disease characterised by liver lesions with infiltrative growth comparable to malignancies. On radiological imaging microcystic honeycomb like lesions are considered characteristic but solid tumors and necrotic cavities (‘pseudocysts’) are also frequently seen which brings solid and cystic differential diagnoses into play[1]. With continuous benzimindazole treatment the 10 year survival rate is excellent for patients with non-curatively resectable AE lesions.

A subgroup of AE patients presents with lesions of the liver hilum. They eventually lead to infiltration and stenosis of major biliary vessels, encasement of vascular structures, lobar atrophy and dilatation of peripheral bile ducts[2–4].

Surgery is the mainstay of AE treatment if the entire parasitic process is resectable with safe distance and has been used as a palliative procedure (bilio-digestive anastomosis) to restore biliary flow[5–10]. More recently endoscopic approaches receive attention. Serial endoscopic dilation with or without placement of plastic stents is a standard tool in the treatment of benign biliary strictures. Furthermore, FCSEMS are increasingly being used in benign biliary conditions such as i.e. strictures and complex bile leaks with the advantage of large calibre and longer duration of patency as well as relative ease of removal[11]. Various approaches in conditions such as post-transplant strictures and primary biliary sclerosis have been described [12–18].

The AE-driven biliary vessel pathology results in variable combinations of stenosis, necrosis and inflammation. Consequently, occlusion of biliary plastic stents due to debris needs to be considered. The objective of this series is to communicate that serial ERC treatment with balloon dilation offers a valuable solution for AE-related biliary pathology.

## Methods

### Clinical Setting and Patients

The Section of Clinical Tropical Medicine at Heidelberg University Hospital runs an interdisciplinary clinic for echinococcosis in cooperation with the Department of Diagnostic and Interventional Radiology with weekly radiological conferences, the Department of Surgery and the Department of Gastroenterology since 1999. Our unit is a national clinical reference center for echinococcosis. The AE patient cohort comprises 106 patients. 80 patients had peripheral and 26 central liver lesions on radiological imaging. Cholestasis was present in in 13/26. Of those 7 patients with cholestasis were treated with ERC, and 6 patients had bilio-digestive anastomosis.

Of this cohort all patients with biliary AE-associated pathology and ERC-D (endoscopic retrograde cholangiography-dilation)/ stenting are reported. The diagnosis of AE was confirmed in accordance with the IWGE-WHO expert consensus criteria[2]. Once AE was diagnosed all patients were started on long-term albendazole treatment and are still under treatment at the time of writing this report.

The following data were extracted from patient notes: age at diagnosis, sex, treatment previous to referral, time from diagnosis to referral, biliary pathology, date of interventions and follow-ups, pre-treatment and latest results of total bilirubin, alkaline phosphatase (AP), CRP, first and maximum balloon size for dilatation, types of stents, post ERC complications cholangitis, bleeding, perforation and pancreatitis.

### Ethics Statement

The Ethical Board of the University of Heidelberg approved (S039/2013) the retrospective analysis of patient data without additional patient consent.

### Intervention

Endoscopic treatment: ERC was carried out using a therapeutic duodenoscope (TJF160R, TJF160VR, TJFQ180V, Olympus Corp., Tokyo, Japan). Selective cannulation of the common bile duct was performed with a guide wire (Jagwire, 0.035 inch, Boston Scientific, Natick, MA, USA, Visiglide, 0.035 inch, Olympus Corp., Tokyo, Japan) or a standard catheter for cases with pre-existing sphincterotomy. All procedures were performed under conscious sedation with propofol and short-acting opiates. All patients received peri-interventional antibiotic prophylaxis.

After visualization of the biliary stricture balloon dilation was performed with 18-Fr balloons at the initial intervention and 24-Fr balloons at subsequent interventions if the stricture was distal to the hilum. If indicated 10 Fr plastic stents (Endoplus Drainage, Pflugbeil, Germany) in appropriate length and number were inserted to bridge strictures.

### Outcome

ERC outcomes were classified as (1) ‘sustained clinical success’: period without ERC-D or stenting for ≥ 6 months after the last endoscopic intervention; ‘assisted clinical success’: period without ERC-D or stenting for < 6 months after the last endoscopic intervention; ‘failure of endoscopic treatment’: persistent stricture and/or cholangitis.

## Results

**Fig 1:**
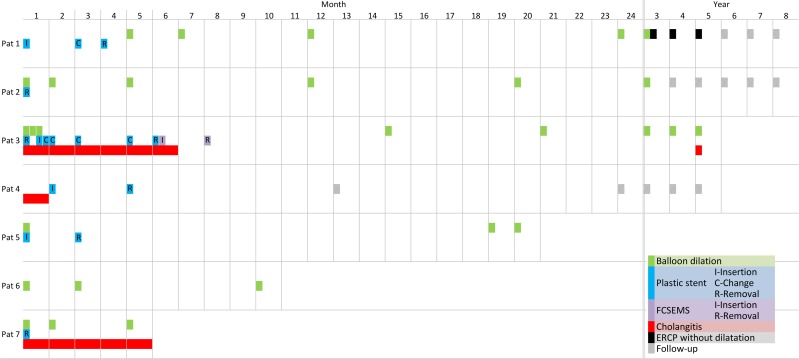
timing of stent insertion and balloon dilation in individual patients. Please note the change of scale at the top of the figure from monthly to yearly after 24 months. At the beginning there is monthly follow-up spacing for 24 months and changes to yearly intervals thereafter. Clinically stable patients are usually followed-up in yearly intervals. Patient 3 shows “sustained clinical success” after FCSEMS removal although biliary duct stenosis is still demonstrable on MRCP (Fig 3). Patient 5 was initially treated in our clinic. In the interval of nearly 2 years without follow-up the patient had non curative liver resection in another hospital.

**Fig 2:**
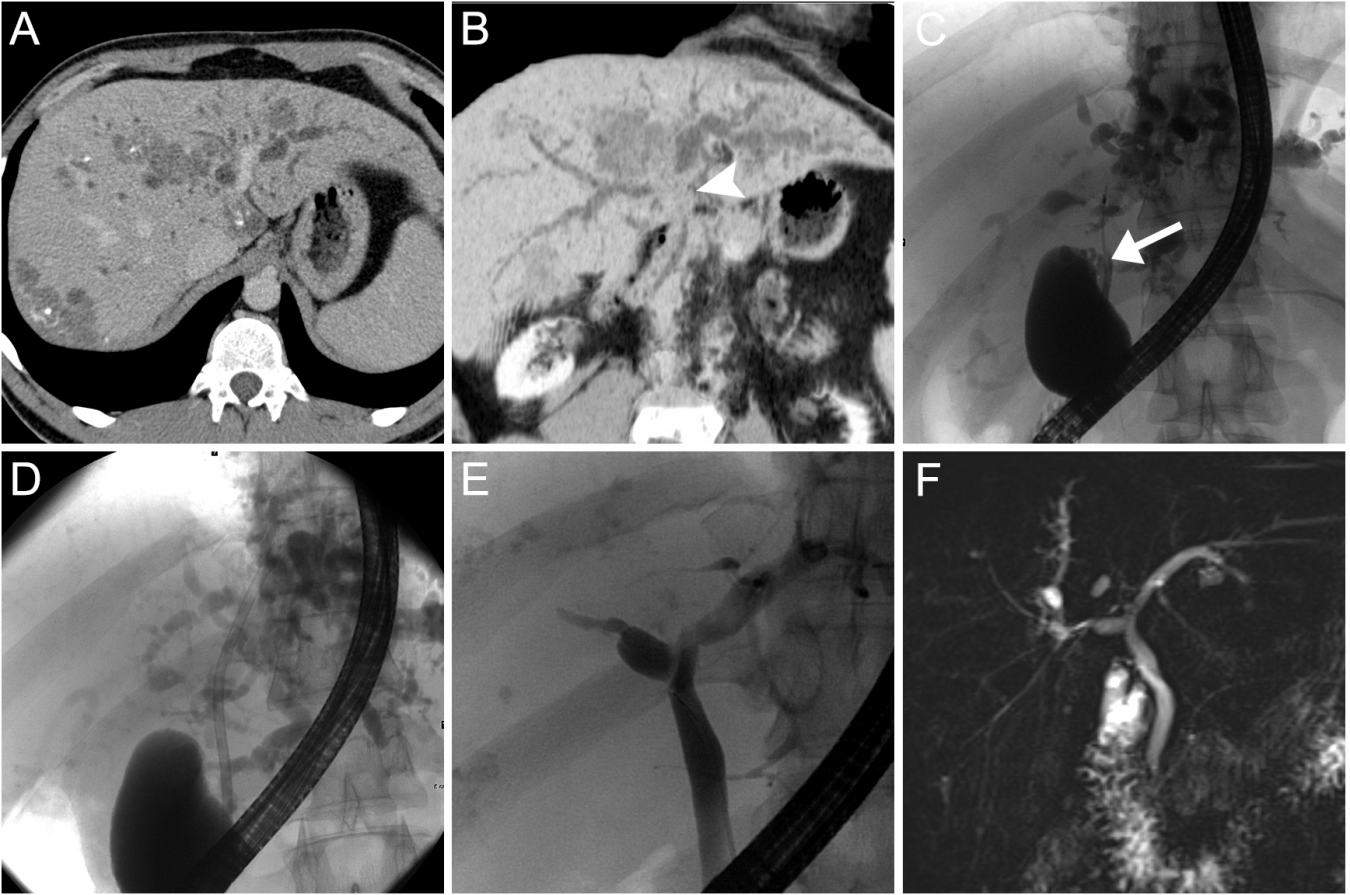
Imaging and ERC findings patient 1. CT scans in axial orientation show multifocal hypovascular calcified mass lesions and obstructive cholestasis with predominance on the left side (A). Minimum intensity projection in oblique coronary orientation visualizes the central occlusion of both bile ducts (B, arrow head). With ERC and initial placement of a plastic stent (which becomes occluded early) and subsequent serial balloon dilation (C, D) patency of bile ducts was regained (E). MRCP confirms sustained clinical success of bile duct recovering (F).

**Fig 3:**
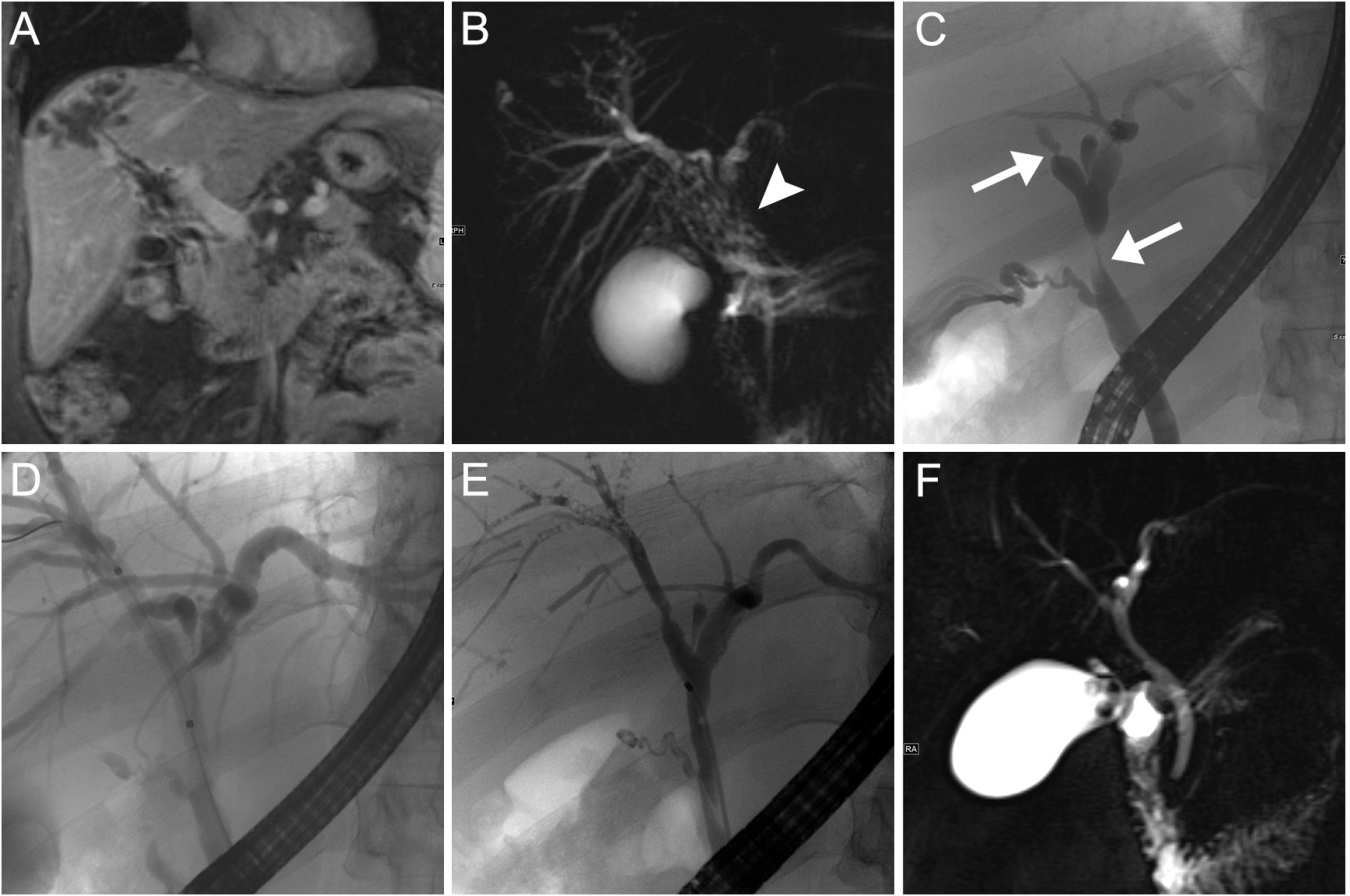
Imaging and ERC findings patient 2. Contrast-enhanced MRI in coronal orientation shows a peripheral liver mass with contiguous perivascular infiltration towards the liver hilum (A). MRCP shows central bilateral bile duct stenosis due to a mass lesion of the liver hilum with micro vesicular appearance (B, arrow head). ERC before (C) und during (D) balloon dilation confirms central stenosis of main and right hepatic ducts (arrows). After serial ERC with balloon dilatation satisfactory remodeling of bile ducts is visible at ERC (E) and MRCP (F).

**Fig 4:**
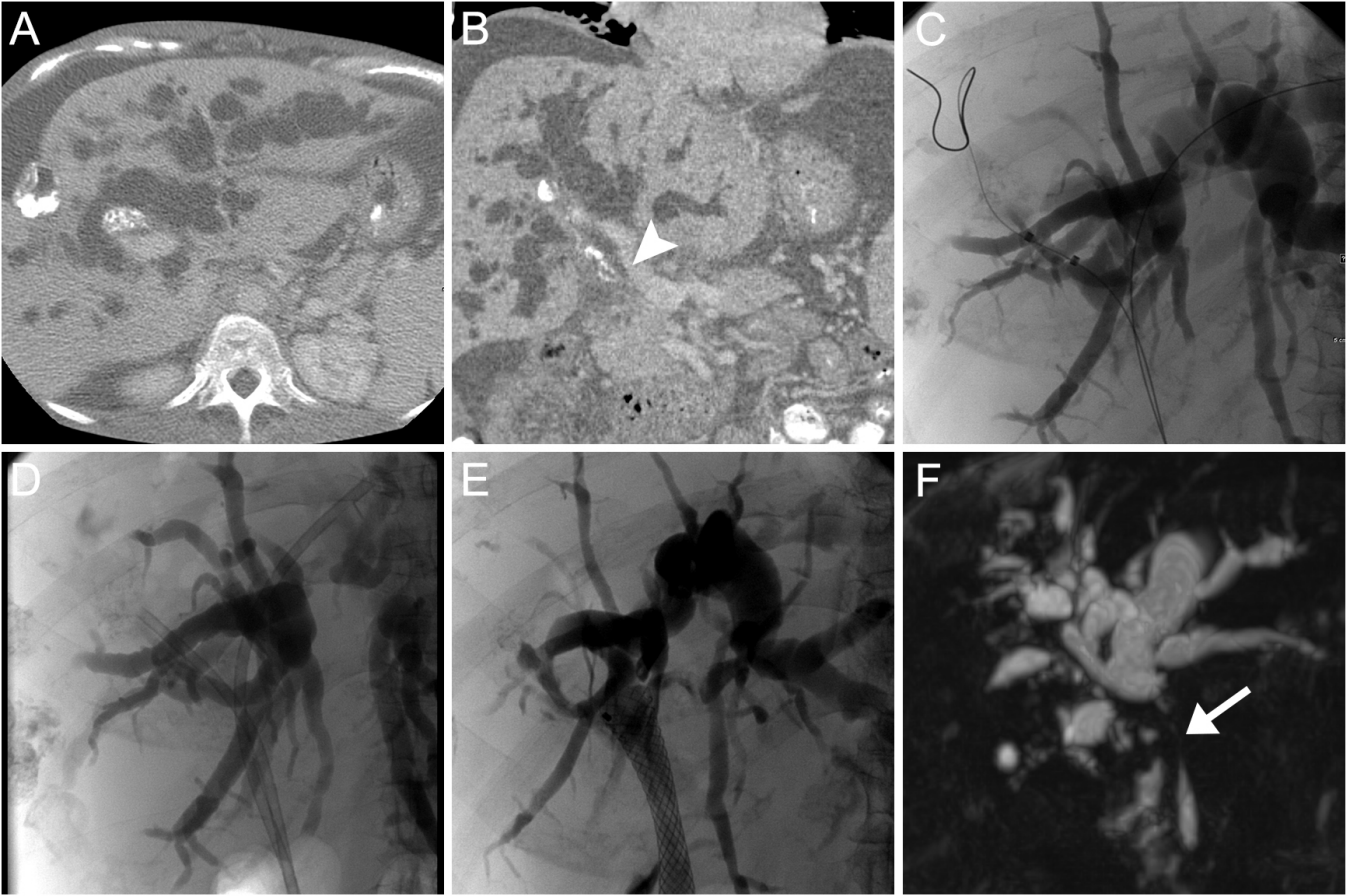
Imaging and ERC findings patient 3. CT scan in axial (A) and coronal (B) orientation and ERC (C) show bilateral obstructive cholestasis with dilatation of intrahepatic bile ducts as well as calcified mass lesions in the periphery and hilum of the liver. Infiltration of the liver hilum with stenosis of the main bile duct (arrow head). Balloon dilation of stenosis and insertion of plastic stents (D). Temporary placement of a FCSEMS (E). MRCP shows persistent stenosis of the main bile duct after extraction of FCSEMS (F) clinically there was”assisted clinical success” (see Fig 1).

Between 2006 and 2015 seven consecutive patients with hepatic AE and biliary infiltration with cholangitis were referred for endoscopic treatment. All patients presented with jaundice. Patient characteristics, pre-referral treatment and biliary duct pathology is summarized in Table 1. Table 2 and Fig 1 summarize laboratory results, types and frequency of interventions, outcome and follow-up. Figs 2–4 illustrate imaging and ERC findings.

**Table 1:** Patient characteristics and treatment complications.

Patient ID	Age at diagnosis, sex	Previous treatment	Time from Diagnosis to center referral	Last follow-up	Total follow-up in months	Biliary pathology on ERC	Post ERC complication	PNM classification
1	32, m	none	Diagnosed in center	5/2015	107	Stenosis of right and left hepatic duct	cholangitis	P4N1M0
2	46, m	ERC with plastic stent	3 months	5/2015	95	Stenosis of common and right hepatic duct	none	P4N0M0
3	45, f	ERC with plastic stent placement,	3 months	12/2014	54	Common hepatic duct destruction, stenosis of right and left hepatic duct	cholangitis	P4N0M0
4	76, m	Albendazole, percutaneous transhepatic cholangio drainage (PTCD)	17 months	2/2014	52	Changes in diameter of common hepatic duct no relevant stenosis	none	P4N1M1
5	43, f	Albendazole, non- curative liver resection	7 years	5/2015	32	Stenosis of common hepatic duct	none	P4N0M0
6	51, m	ERC with plastic stent placement, explorative laparotomy, Albendazole	20 months	12/2014	13	Stenosis of common hepatic duct	none	P4N0M0
7	73, f	Albendazole, ERC with plastic stent placement	4 weeks	3/2015	6	Stenosis of common hepatic duct	none	P4N1M1

**Table 2:** Diagnostic and treatment data.

Patient ID	Pre total bilirubin (μmgol/l)	Last total bilirubin (μmol/l)	Pre AP (U/l)	Last AP (U/l)	Pre-treatment CRP (mg/l)	Last value CRP mg/l	Balloon size first ERCP (Fr)	Maximum balloon size (Fr)	Use of stent	Therapeutic success	Total number of ERCPs
1	71.4	18.7	623	45	3	<2	18	24	EP	sustained	11
2	88.4	8.5	187	82	99	<2	18	24	-	sustained	6
3	141.1	47.6	790	282	205	28	18	24	EP, FCSEMS	assisted	15
4	176.8	13.6	-	397	14	21	n.a.	n.a.	n.a.	sustained	3
5	282.2	17.0	171	88	8	5.9	18	18	EP	assisted	4
6	108.8	15.3	-	55	-	2.6	18	24	-	assisted	3
7	192.1	78.2	632	522	399	92	18	24	-	assisted	3

EP: endoprosthesis, BD: Balloon dilation, FCSEMS: fully covered self-expandable metal stent, n.a. not applicable

## Discussion

Alveolar echinococcosis (AE) of the biliary tree is characterised by destruction of biliary vessels due to infiltrative, malignancy like growth of AE liver lesions. In general, growth of AE can be halted with albendazole, a benzimidazole with parasitostatic effect. ERC is the treatment of choice for benign biliary strictures and general ERC treatment recommendations are being applied in AE patients [11]. Our series shows that serial endoscopic balloon dilation and stenting combined with benzimidazole treatment can re-establish and maintain biliary duct patency for many years. Thus, interventions with a more profound impact on the quality of life such as percutaneous bile drainage can be avoided or postponed. Well established ERC management for AE patients could possibly even postpone liver transplantation which is highly problematic in this entity as immunosuppression favors re-growth of non-resected or non-resectable AE components and spread with distant metastases.

We have observed stent occlusion of plastic stents in two patients. Of which in one repeated stent occlusion is reflected by the high frequency of stent changes at the beginning of ERC treatment. The problem of stent occlusion is also mentioned in the current expert consensus guidelines[2] and may be increasingly so in AE patients due to inflammation and necrotic debris production. This favours the concept of balloon dilation alone with restrictive policy of stent placement. Although biologically and technically plausible the limited number of patients does not yet allow drawing final conclusions on the preferable endoscopic technique. In one patient sustained success could not be achieved despite repeated insertion of plastic stents and balloon dilation but with temporary insertion of a FCSEMS, an evolving technique in benign biliary disease [11].

Our report aims at proof of concept as a first step in further research of endoscopic treatment of AE patients [19, 20].

Innovative management strategies for AE patients with cholestasis not eligible for curative surgery are desperately needed. There is no published evidence to support the current recommendation of percutaneous drainage as an equivalent treatment alternative to ERC management [2]. Repeated balloon dilation is in accordance with current ERC guidelines and appears a promising first line management of biliary obstruction. This approach is in accordance with current ERC guidelines[11] and in general minimally disruptive for patients.
